# Variation in early number skills and mathematics achievement: Implications from cognitive profiles of children with or without Turner syndrome

**DOI:** 10.1371/journal.pone.0239224

**Published:** 2020-10-02

**Authors:** Sarah L. Lukowski, Emily R. Padrutt, Kyriakie Sarafoglou, Judith L. Ross, Jennifer R. Law, Rachel E. Olson, Michèle M. M. Mazzocco

**Affiliations:** 1 Institute of Child Development, University of Minnesota, Minneapolis, Minnesota, United States of America; 2 Department of Pediatrics, University of Minnesota Medical School, Minneapolis, Minnesota, United States of America; 3 Department of Experimental and Clinical Pharmacology, University of Minnesota College of Pharmacy, Minneapolis, Minnesota, United States of America; 4 Department of Pediatrics, Nemours DuPont Hospital for Children, Thomas Jefferson University, Philadelphia, Pennsylvania, United States of America; 5 Division of Pediatric Endocrinology, Department of Pediatrics, University of North Carolina at Chapel Hill, Chapel Hill, North Carolina, United States of America; French National Center for Scientific Research (CNRS) & University of Lyon, FRANCE

## Abstract

Individuals with Mathematics Learning Disabilities have persistent mathematics underperformance but vary with respect to their cognitive profiles. The present study examined mathematics ability and achievement, and associated mathematics-specific numerical skills and domain-general cognitive abilities, in young children with Turner syndrome compared to their matched peers. We utilized two independent peer groups so that group comparisons would account for verbal skills, a hypothesized strength of girls with Turner syndrome, and nonsymbolic magnitude comparison skills, a hypothesized difference of girls with Turner syndrome. This individual matching approach afforded characterization of mathematics profiles of girls with Turner syndrome and girls without Turner syndrome that share potential key features of the Turner syndrome phenotype. Results indicated differences in mathematics ability and nonsymbolic magnitude comparison tasks between girls with Turner syndrome and peers with similar levels of verbal skill. Mathematics ability and mathematics achievement scores of girls with Turner syndrome did not differ significantly from their peers with similar levels of accuracy on a nonsymbolic magnitude comparison task. Cognitive correlates of mathematics outcomes showed disparate patterns across groups. These quantitative and qualitative differences across profiles enhance our understanding of variation in mathematics ability in early childhood and inform how mathematics skills develop in young children with or without Turner syndrome.

## Introduction

Mathematics learning disabilities (MLDs) are heterogeneous, but they are similar to each other in terms of their persistence throughout life and the presumed biological nature of their diverse underpinnings [1]. MLDs and other mathematics learning difficulties are collectively associated with individual differences across a wide array of skills, including those related to domain-specific (e.g., numerical processing skills) and domain-general (e.g., language and executive function [EF] skills) cognition. Therefore, profiles of cognitive strengths and differences of individuals with MLD vary widely. This variation poses challenges to determining appropriate instructional supports and interventions.

Research in mathematics cognition has long posited that meaningful subgroups of MLD can be delineated based on cognitive profiles [2–5], and that profiles across individuals with different degrees of severity in mathematics underachievement differ qualitatively [6]. A better understanding of these subgroup differences may inform approaches to individualize instruction or support alternative strategies for different learners. Unfortunately, the field is lacking well-established universal subgroup classifications of MLD, which leads to inadvertently collapsing across subgroups in research samples and–potentially–in practice. These collapsed data in turn lead to group-level findings that may mask important subgroup differences [6]. In this study, we draw from findings that some of the liability for risk of MLD derives from biological factors [7, 8], and we consider a specific condition that confers biological risk for MLD: Turner syndrome (TS). TS is a sporadic monosomy disorder that results from either complete or partial loss of one of two X chromosomes typically present in girls and women [9]. We build upon prior research on mathematics underperformance in individuals with TS to explore the relation between select features of the TS cognitive phenotype, including numerical processing skills and low mathematics achievement in children with or without TS during the early school years. We adopt this approach as a means to potentially identify differences in MLD profiles across young children with or without TS.

Prior studies of TS have consistently shown that girls and women with TS are more likely to meet criteria for MLD compared to persons from the general population [10, 11]. Across studies, approximately 55–70% of girls with TS meet criteria for MLD on a least one assessment (e.g., [11, 12]), in contrast with 26–33% of peers in matched comparison groups in the same studies [11, 12] and with the reported incidence rate of approximately 6–10% of the general population based on more stringent MLD criteria [13]. Mathematics differences observed in girls with TS cannot be attributed to low intellectual ability, because general intellectual functioning is typically not affected by TS [14]. Moreover, girls and women with TS tend to outperform their peers on verbal abilities such as vocabulary and reading skills [15, 16]; and within group, Verbal IQ scores among girls with TS tend to outpace their own Performance IQ-related skills (see [14] for review). The primary aim of the present study was to examine profiles of domain-specific (i.e., number- and mathematics-oriented) and domain-general (i.e., those that underlie multiple academic domains) cognitive skills, characterizing the relation between these cognitive skills and mathematics achievement outcomes and how these relations vary across children with TS and children that share a specific feature of the TS cognitive phenotype.

### Why study early mathematics skills in girls and women with TS?

Reviews of the TS cognitive phenotype frequently include reference to early mathematics differences, whether in summaries directed to families [17], practitioners [18], or researchers [19]. However, nuances of the early TS mathematics profile are difficult to discern from earlier studies of TS, because participant samples in prior studies were typically small or comprised of individuals that spanned wide age groups. Moreover, nearly half (42%) of those studies included only participants older than 8 years of age; see S1 Table for prior studies. To our knowledge, only three studies of mathematics difficulties in children with TS focused exclusively on the early school years (through Grade 3), and those studies involved a single longitudinal sample [11, 20, 21]. Expanding this limited research base is important for determining the early profile of MLD in TS, aiding in future identification of risk for MLD and, potentially, supporting early prevention or intervention efforts.

Accordingly, one goal of the present study was to ascertain a sample of young girls with TS within a fairly narrow age span representative of the early childhood period (4 to 8 years old), and to identify whether the relatively higher rate of mathematics underperformance associated with TS during the early school years replicates in this novel sample. Toward this goal, we assessed the TS cognitive phenotype using a battery of measures administered to girls with or without TS, with a focus on how numerical skills and other cognitive correlates of mathematics outcomes vary between these groups. Our assessment battery was informed by prior findings of numerical processing difficulties in adolescents and adults with TS [10, 22], despite additional evidence that standardized mathematics achievement scores of girls with TS typically increase from elementary to middle school [21]. The confluence of mathematics achievement improvement and persistently low number skills in girls with TS is atypical in the general MLD literature, and raises questions as to *why* girls with TS show such improvement in mathematics achievement when numerical processing difficulties persist [21], and if that alleged improvement reflects differences in relations between numerical processing and mathematics in children with or without TS. One possible explanation for the observed improvement in mathematics achievement is reliance on compensatory processes to overcome numerical processing difficulties. Accordingly, in addition to our focus on numerical processing and mathematics achievement, in the present study we focused on verbal skills as a potential compensatory process on which girls with TS may rely to overcome their presumed persistent numerical difficulties; and we therefore based a comparison group on girls individually matched to girls with TS on verbal skills. We also focused on EF skills because of the importance of EF skills in early mathematics achievement in the general population, including during early childhood [23]. Rather than create a comparison group matched on EF skills, we compared the relation between EF and mathematics skills within each group, because EF skills are highly variable in girls with TS [24]. To the extent that the TS phenotype broadly pertains to other children with similar cognitive phenotypes, the findings from our study may inform general risk of MLD among persons who share these cognitive characteristics.

In the following sections, we describe three cognitive domains typically addressed in both the MLD literature and the literature on the TS cognitive phenotype: mathematics, verbal, and EF skills. Included under the domain of mathematics skills, we also describe a novel construct–children’s resolution of lexical ambiguity specific to number words [25]–as a potential correlate of mathematics outcomes in girls with TS. We predicted that mathematics outcomes in girls with TS may coincide with numerical processing differences and with verbal strengths or widely ranging EF skills also associated with TS. We aimed to identify whether correlations among these skills differ when examined among children with TS compared to children without TS from a matched-comparison group, which would suggest group differences in the skills or strategies that support young children’s early mathematical thinking or achievement.

### Mathematics skills

The evidence for a heightened risk for MLD in girls with TS is drawn from research spanning individuals ages 5 years to adulthood. Based on a meta-analysis of 17 such studies, Baker and Reiss [26] reported moderate to large effect sizes when comparing girls and women with TS to their peers on standardized (effect size; ES = 1.221) and non-standardized (ES = 0.562) measures of mathematics. This effect emerged for accuracy (ES = 0.339) and speed (ES = 1.719) on mathematics-related measures. Similarly, the effect size was large across all kinds of calculation problems, including arithmetic and geometry (ES = 1.099); but only modest for problems primarily reflective of foundational numerical skills such as subitizing, counting, or reading and writing numbers (ES = 0.101). Although five of the 17 studies reported in their meta-analysis included at least some participants under 8 years of age, only one study [20] focused exclusively on girls under 8 years of age. Indeed, the ages represented in this meta-analysis reflect the trend across the literature which vastly under-represents the mathematics profiles of young girls with TS. Still, there is clear consensus that mathematics ability is below average in a significant proportion of girls and women with TS from early childhood to adulthood.

Beyond broad mathematics ability and achievement levels, there is considerable interest in the role that basic numerical processing skills play in girls with TS. Numerical skills include nonsymbolic magnitude comparison (MC_N_) skills that reflect an ability to represent and compare quantities, such as two sets of dot displays, independent of the words and symbols used to convey those quantities. Although there is considerable debate concerning what skills underlie performance on MC_N_ tasks [27–29] and the influence of task demands on MC_N_ task performance, there is general agreement that these skills differ from symbolic magnitude comparison skills that rely on digits and number words. Researchers also generally agree that symbolic magnitude comparison skills account for more variance in mathematics than do their nonsymbolic counterparts [30], particularly when predicting highly symbolic mathematics skills such as fractions [31]. Still, MC_N_ represents a core numerical skill related to individual differences in mathematics [32] during the early school years [33, 34], and may be particularly relevant as an earlier predictor of MLD [34].

Whether MC_N_ skills contribute to the TS profile is an unresolved question, and to our knowledge this question has not been addressed in any study of young girls with TS. Although one study found no differences between young girls with TS and their peers on a paper-and-pencil MC_N_ task [11], the paper format of the task cannot prevent reliance on counting or other symbolic processes, and thus provides insufficient evidence of intact MC_N_ skills in TS. More recently, Attout, Noel, Nassogne, and Rouselle [35] administered several computerized MC_N_ tasks to girls and women with TS, and varied the tasks in order to isolate contributions of visual-spatial and short term memory demands. Importantly, their mixed-age group of 20 girls and women with TS included only six children, none under 7 years of age (all were 7 to 12 years old); and also included five adolescents and nine adults who were 20 to 33 years of age. Although their study does not address the TS cognitive phenotype in early childhood, their tasks were carefully controlled and revealed specific conditions under which their participants with TS were less precise than peers in their MC_N_ judgments. Specifically, in their study, participants with TS did not differ from their peers when MC_N_ displays were viewed simultaneously (side-by-side), as occurs in the vast majority of MC_N_ tasks used by researchers [e.g., 32]; moreover, there was no evidence that visual-spatial abilities drove performance in the TS group. On average, the group with TS performed less accurately than their peers when pairs of MC_N_ displays were viewed sequentially (i.e., across two computer screen shots) separated by a short time delay, or when pairs of MC_N_ auditory stimuli were presented sequentially (also with a delay), suggesting a role of working or short term memory. These findings raise questions about the numerical skills involved in MC_N_ among persons with TS and the developmental changes in cognitive correlates underlying those skills in girls and women with or without TS. By including broad measures of mathematics outcomes and MC_N_ as a potential correlate of mathematics outcomes in the present study, we were able to evaluate the role of MC_N_ in mathematics performance among young girls with TS relative to their peers. By including a peer group matched to girls with TS on MC_N_ accuracy, we tested the hypothesis that, among individuals with poor MC_N_ accuracy, verbal skills (a relative strength in the TS phenotype) correlate more strongly with mathematics outcomes in children with TS relative to children without TS. We based this prediction on evidence of potential compensatory skills associated with verbal strengths in girls with TS, as elaborated in the next section.

Our interest in the relation between mathematics and verbal skills in TS led us to include another experimental task at the intersection of these two domains, which pertains to deciphering the meaning of numerical words based on context. Mazzocco, Chan, and Sera [25] proposed number word interpretation as a novel aspect of number knowledge, particularly in contexts where numerical meaning is ambiguous. This notion of numerical lexical ambiguity has been explored in adults [36] and preschoolers [25], but it has not been previously investigated in girls with TS. Accordingly, we included an experimental measure of numerical ambiguity based on the notion that number words are inherently confusing in a manner similar to homonyms and other forms of lexical ambiguity. Earlier work on this notion exploited previous research on other forms of lexical ambiguity and revealed individual differences in the extent to which children demonstrated literal interpretation response biases [25]. For instance, in earlier studies of children’s interpretation of non-numerical homonyms, researchers identified developmental variation in children’s tendencies to rely on verbal context to differentiate a novel, secondary meaning of a homonym (e.g., bats are flying mammals) from a more familiar meaning (e.g., wooden baseball bats) such that literal response biases were evident in 4- and 5-year-olds, but diminished by age 10, with large individual variation in this response bias at ages 7 or 8 years [37, 38]. Similarly, in the face of ambiguous comparisons of quantity (e.g., which is more, four cookies or two bags of cookies?), some preschoolers showed a bias towards associating a larger number word with the more numerous set of items, even in the presence of visible conflicting evidence (e.g., two bags containing a total of six cookies) [25]. Accordingly, we used an investigator-designed measure of numerical ambiguity among our measures of mathematics-oriented and number knowledge skills, and proposed that, in girls with TS, performance on this measure may be an indicator of verbal compensatory strategies and therefore related to general levels of mathematics achievement.

### Verbal skills

Earlier reports that girls and women with TS had average or above-average verbal skills [14, 39] were typically based on higher Verbal compared to Performance IQ scores [40–43] and later delineated more specifically. For instance, the TS cognitive phenotype was described as including specific strengths in phonological processing and vocabulary [14–16, 39], average receptive and expressive vocabulary skills in young girls with TS ages 5 to 12 years [44], and a receptive vocabulary advantage in girls ages 9 to 12 years [16]. The verbal skills of interest in the present study, therefore, concern verbal comprehension and expressive vocabulary skills, which we propose may support emerging mathematics skills. For instance, evidence from an fMRI-based problem verification study indicated that girls with TS may have relied on verbal strategies to evaluate arithmetic problems [45]. In that study, girls with TS were as accurate as their peers in evaluating the veracity of solutions to mathematics problems, but they showed increased activation of the temporal lobe, an area of the brain associated with verbal processing, during the task; and this neural response pattern differentiated the girls with TS from their peers. Accordingly, in the present study we explored relations between verbal abilities, numerical processing, and mathematics ability and achievement in children with or without TS in the early school years.

### Executive function skills

Difficulties with EF skills have received considerable attention in both the TS and educational psychology literatures because of their theoretical and empirical [46, 47] relevance to academic achievement in general and mathematics achievement specifically. A meta-analysis of 14 studies of EF in girls with TS [24] found that the TS cognitive phenotype was associated with underperformance on EF measures (mean Hedge’s g = -0.67), but that the effect size between children with TS and their matched peers ranged widely depending on whether the core component of EF being measured in a given study was working memory (range Hedge’s g: -1.17 –-0.21), cognitive flexibility (range Hedge’s g: -1.54–0.26), or inhibitory control (range Hedge’s g: -1.01–0.08). However, differentiating these components in young children is problematic because factor analyses do not support a three-factor structure of EF until around age 15 years [48]. Accordingly, EF researchers often use tasks that simultaneously tap several components of EF in their work with young children, and acknowledge that these composite measures of EF may not be sufficiently sensitive to pinpoint distinct EF components. In the present study, we relied on two such composite measures of EF.

A few researchers have relied on composite measures to examine EF skills of younger children with TS. For instance, Kirk, Mazzocco, and Kover [49] reported that 8- to 9-year-old children with TS scored lower than their matched peers on the Contingency Naming Task (a Stroop-like task that requires naming the color or shape of individual objects), which involved ignoring one feature or the other, according to rules that changed across trials (requiring cognitive flexibility), and doing so under increasing working memory demands. The group discrepancies reported by Kirk et al. [49] increased from Grades 1 through 7, as reported in a longitudinal follow up study with this sample [21]. These findings suggest that the development of EF skills in girls with TS may not parallel that of typically developing children, and it may follow that the relation between EF and early mathematics in girls with TS also differs from that of their peers. Given the relation between EF skills and early mathematics skills in general and the wide-ranging EF skills reported in girls with TS, in the present study we examined individual differences in EF skills as one possible cognitive correlate of mathematics learning difficulties in girls with or without TS.

### Purpose and hypotheses of the current study

The purpose of the current study was to examine the cognitive phenotype of young girls with TS as it pertains to their mathematical skills. By focusing our study on girls enrolled in prekindergarten to Grade 3, we were better able to characterize the cognitive phenotype of TS during the early school years, a period during which early identification of (and intervention for) early mathematics difficulties may be critical. We anticipated that mathematics ability and achievement scores of girls with TS would be in the below average to low-average range, consistent with the prior literature [12, 20, 44]. Although MC_N_ skills have not been previously considered in young girls with TS, we hypothesized that girls with TS would have weaker MC_N_ skills compared to peers, based on the evidence of persistent numerical processing difficulties described earlier (e.g., [10, 21]). There is no prior literature on children’s resolution of ambiguity in number words in children with TS, and our inclusion of this construct and its contribution to numerical processing in TS was exploratory, as a possible extension of reported verbal strengths associated with TS.

We also hypothesized that girls with TS would, on average, have average to high-average verbal skills, consistent with verbal strengths reported for girls and women with TS at all ages (e.g., [16, 44]). This may lead young girls with TS to have a verbal advantage over matched peers with similar levels of MC_N_ skills, and we proposed that verbal skills would be positively correlated with mathematics achievement to a greater degree among girls with TS relative to their peers. Prior studies on EF skills in girls or women with TS suggest widely variable performance on measures of EF compared to peers, and we hypothesized that this pattern would extend to younger girls with TS and their peers.

## Methods

### Ethics statement

The study protocol was reviewed and approved by the Institutional Review Board at the University of Minnesota. All parents provided written informed consent for their child to participate. Children 8 years of age or older also provided written informed assent, in accordance with University Institutional Review Board policies.

### Participants

#### Participants with Turner syndrome

Girls with TS were recruited by distributing recruitment flyers to the coordinators of Turner syndrome clinics or support groups across the continental United States. Coordinators were asked to distribute flyers to parents of girls who were 4 to 8 years old and enrolled in prekindergarten to Grade 3, or who would meet these criteria within the study timeline.

Parents of 70 children requested more information about the study and completed our inclusionary criteria screening based on karyotype and general background review. Of these, 10 potential participants were excluded because the child’s karyotype was associated with general intellectual disability, or because the karyotype revealed another chromosomal abnormality in addition to TS. Ten additional potential participants were excluded based on their developmental or neuropsychological diagnoses or history of major medical interventions with possible cognitive implications. Of 50 children who were eligible to participate based on our enrollment criteria, 44 children from 22 states enrolled in the study and were included in the current analyses. The six additional participants who were eligible and scheduled to participate ultimately cancelled due to stay at home orders associated with the COVID-19 pandemic. Of the 44 who participated, 24 children had a diagnosis of classic TS (45,X) and the remaining 20 children had a mosaic karyotype. At time of testing, the 44 children ranged in age from 4 years, 8 months to 8 years, 11 months (*M* = 6 years, 10 months; *SD* = 16.59 months), and were attending (or recently completed) prekindergarten (*n* = 11), kindergarten (*n* = 7), Grade 1 (*n* = 10), Grade 2 (*n* = 10), or Grade 3 (*n* = 6).

#### Comparison group composition

To create two independent comparison groups, we individually matched girls with TS to girls drawn from a larger ongoing study of early mathematics conducted in an urban public school district. Recruitment for this larger study occurred at school events, through mailings distributed via classrooms, or through a university participant pool of families within the same postal codes as the participating schools. Across the 3 years of recruitment for the larger study, 360 children ultimately participated (*n* = 171 girls).

From this group of 171 girls, we identified those individually matched to a participant with TS on verbal or MC_N_ accuracy scores and on age within 4 months when possible. To the extent possible, we also matched on grade level and school months, defined as months of schooling completed since kindergarten entry (i.e., the onset of formal schooling) based on a 9-month school year. All prekindergarten participants were assigned 0 school months. This approach allowed us to create two independent comparison groups, each based on one aspect of the TS profile that we hypothesized was relevant to MLD, while also matching on age and months of formal schooling completed to the extent possible. Thereafter, if more than one potential matched pair was available, we considered child race/ethnicity, parental education, child eligibility for free or reduced-price meals at school, and regular exposure to a language other than English in the home as additional matching criteria, although it was not possible to match all pairs on all criteria. In total, 88 girls comprised the two comparison groups (44 per group).

#### Matched pairs

All participants were girls. Of the 88 participants across two comparison groups, all but five (94.31%) were individually matched to a girl with TS on grade; the exceptions involved four girls with TS who completed the study during July through September. This includes one kindergartner with TS whose age- (within four months) and school-months- (within four months) Verbal- and MC_N_-matched peers were both in first grade; two second graders with TS, one whose closest age- (within four months) and MC_N_-matched peer was in first grade and the other whose age- (within four months) and school-months- (within four months) MC_N_-matched peer was in third grade; and one third grader with TS whose closest MC_N_-matched peer was in second grade.

Across the Verbal- and MC_N_-matched pairs, 90.9% and 70.5% were matched on school months within 4 months, respectively, and 100% were matched on school months within 7 or 12 months, respectively.

Among Verbal-matched pairs, 93.2% were matched on age within 4 months (among those with age spans greater than 4 months, one girl with TS was 7 months younger and two girls with TS were 8 or 9 months older than their matched peer). Among the MC_N_-matched pairs, 63.6% were age-matched within 4 months and the remaining pairs were age-matched within 13 months (among those with age spans greater than 4 months, six girls were younger than their peer by 5–9 months and 10 girls were older than their peer by 5–13 months).

Based on a demographic questionnaire completed by parents of all girls with TS and 82 matched peers (43 from the Verbal-matched group), just over 70% of pairs were matched within 2 years on highest parent education (75.0% and 70.5% for the Verbal- and MC_N_-matched pairs with reported education levels, respectively), and about 50% were matched on exposure to secondary language(s) in the home or by family members (54.5% and 47.7%, respectively) and over 50% were matched on free or reduced-price meals eligibility (70.5% and 52.3%, respectively).

#### Participant summary

Table 1 is a summary of the demographic characteristics for participants with TS and participants in the individually-matched comparison groups.

**Table 1:** Descriptive summary of demographic characteristics across three participant groups.

Variable	TS	Verbal-Matched	MC_N_-Matched
Age (yrs)			
*M (SD)*	6.86 (1.38)	6.84 (1.31)	6.75 (1.20)
Range	4.67–8.92	4.83–8.92	4.75–8.83
School months			
*M (SD)*	13.55 (11.22)	13.20 (11.00)	13.32 (10.79)
Range	0–36	0–34	0–34
Parent education (yrs)			
*M (SD)*	16.72 (1.96)	16.88 (1.45)	15.95 (2.66)
Range	12–18	12–18	8–18
Race/ethnicity (%)			
American Indian	0	2.2	0
Asian	0	11.4	15.9
Black	2.3	2.2	22.7
Hispanic or Latino	9.1	4.5	4.5
Multiracial	15.9	13.6	18.2
White	72.7	61.4	27.3
No response	0	4.5	11.4
Other language in home (%)	11.4	32.6	51.2
FRM-eligible (%)	13.6	29.3	51.2

TS, Turner syndrome; MC_N_, nonsymbolic magnitude comparison; FRM, free or reduced-price meals.

### Measures

#### Mathematics skills

We administered two standardized measures of mathematics ability or achievement that yield age-referenced standard scores and total raw scores that were our primary *outcome* variables of interest. We also administered two experimental measures of numerical processing, which provided domain-specific scores we tested as potential correlates of mathematics outcomes.

The *Test of Early Mathematical Ability*, *Third Edition* (TEMA-3; [50]) is a standardized measure of formal and informal mathematics skills designed for use with children ages 4 to 8 years. It is widely used by researchers of early mathematical thinking. Items concern informal numbering, number comparisons, calculations, and concepts; and formal numeral literacy, number facts, calculation, and place value. Tasks within the TEMA-3 involve counting, story problems, and solving paper-and-pencil or mental calculations. The TEMA-3 has very good internal reliability (α = .94; [50]). We reported the participants’ age-normed mathematics ability scores (*M* = 100, *SD* = 15) and examined total raw scores (out of 72) as our outcome variables of interest.

The *Woodcock–Johnson Achievement Test*, *Third Edition* (WJ-III [51]) is a standardized measure of academic achievement designed for use with individuals aged 2 to 90 years. It is widely used by practitioners and researchers. We selected the Applied Problems subtest (WJ-III AP), during which an examiner reads problems aloud while showing participants relevant stimuli. Participants listen to the examiner, identify what information is relevant or irrelevant to generate a response, determine the appropriate procedure to carry out, and generate or calculate a solution. Early problems require pointing or verbal responses, and later items may involve paper and pencil solutions. The WJ-III AP subtest has very good median internal reliability for individuals in our participants’ age range (α = .92; [52]). We reported age-normed scores (*M* = 100, *SD* = 15) and examined total raw scores (out of 63) as our outcome variables of interest.

The *Psychological Assessment of Numerical Ability* (Panamath; [32]) is a computerized task widely used by researchers to assess nonsymbolic approximate magnitude comparison of large (i.e., ≥ 5) numerosities. We used the version available at Panamath.org, which is appropriate for individuals ages 3 to 85 years, administered by an examiner on a 13-inch MacBook Air laptop computer. This version has a reported split-half reliability of α = 0.72 in a sample of prekindergarten children [33] and 11- to 85-year-old participants [53]. During the standard administration of the Panamath task appropriate for our participant age group, per test trial, participants simultaneously view two arrays of dots. These arrays appear on separate sides of a computer screen for a brief, fixed interval; each trial is followed by a backward mask. The limited duration of stimulus exposure and the backward mask both serve to prevent children from relying on counting to respond. Per trial, participants are asked to indicate which side of the screen has more dots. Trials conform to one of the two following conditions: Half of the trials are controlled for surface area, and half are controlled for average dot size, in order to prevent responses based on either of these features rather than numerosity. These controls are important ways to evaluate precision across different ratios in numerosity and visuo-spatial features of a set. Within each condition, individual trials vary systematically in terms of the ratio bin by which the set size between the two arrays differs, and item difficulty increases as ratios approach 1.

Default or advanced Panamath settings can be used to determine interval duration and duration of the backward mask. As the initial study of MC_N_ skills in young girls with TS, we were interested in determining the accuracy with which participants would compare set sizes within a range of ratios that included very easy to moderate discriminations. We therefore fixed settings so that each participant completed the same two practice trials and the identical set of 88 test trials, appearing in the same order, for 2128 ms per trial each followed by a 200 ms backwards mask. We set ratio bin sizes to 1.3, 1.5, 1.8, and 2.8. We used percent accuracy as a variable of interest across all trials, as is recommended for this age group [54] but also examined accuracy across conditions and ratio bins. Interpretations of MC_N_ performance are generally evaluated at the ratio bin level, with accuracy at or above 75% per ratio bin reflecting an ability to reliably discriminate quantities that differ at that particular ratio bin. Since all our ratio bins were constant across all participants, we examined total percent accuracy, with an expectation of age-appropriate increases from age 4 to 8 years. However, we selected ratio bins we believed would avoid ceiling performance, and our results confirm this assumption.

The *Numerical Ambiguity Interpretation Task* (NAIT, [25]) is an examiner-created task designed to investigate children’s ability to navigate the lexical ambiguity associated with number words in context (e.g., whether “four muffins” refers to more or fewer muffins relative to “three bags of muffins”). Following a warm-up, during each of 48 trials the participants listen to the examiner read a sentence, view a set of corresponding pictures, and answer the experimenter’s prompt for which child had more. There are six item types utilized to probe for different levels of ambiguity; see [25] for detailed descriptions. A key feature of the task is whether children make literal interpretations of number words (e.g., four is greater than three) without considering contextual evidence to the contrary, in which case they may respond incorrectly on half of the trials (e.g., by indicating that a child with four muffins has more muffins than a child with three bags of muffins despite visual evidence that the bags contain a total of six muffins). Total number of items (out of 24) on which a child’s incorrect response was consistent with this large number word bias error (LNWB) was used in analyses.

#### Verbal knowledge

We administered two widely used clinical measures of receptive and expressive verbal knowledge, a domain-general correlate of interest because it is a reported area of strength in studies of the TS cognitive phenotype.

The *Kaufman Brief Intelligence Test*, *Second Edition* (KBIT-2; [55]) is a standardized measure of intelligence designed for individuals ages 4 to 90 years. It is widely used by researchers and practitioners as a rapid estimate of IQ. We administered the Verbal Knowledge subtest (KBIT-2 VK), which taps general information knowledge and receptive language. Per trial, participants view an array of six illustrations or photographs while the examiner prompts with questions that range from item identification (e.g., “Point to grocer”) to general information (e.g., “Point to what shows you the temperature”). The reliability of the KBIT-2 ranges from 0.74–0.87 for the ages of participants in the present study [55]. We reported the participants’ age-normed scores (*M* = 100, *SD* = 15) and used raw scores in subsequent analyses.

The *Boston Naming Test*, *Second Edition* (BNT-2; [56]) is a measure of confrontational picture naming widely used by neuropsychology practitioners and researchers; it is the naming task most widely used by pediatric neuropsychologists [57]. During administration each participant views line drawings while an examiner prompts the participant to name each one. Following this protocol, in the present study, the examiner also prompted incorrect responses that were close associates (e.g., naming a soup pot a soup cooker) unless an incorrect response was clearly an error (e.g., examiner did not prompt if the soup pot was called a frying pan). We removed one BNT-2 item due to its cultural insensitivity, resulting in a maximum total score out of 59. To our knowledge, reliability information for children’s completion of the BNT-2 has not been published, but internal reliability for the BNT in adults is adequate; α = .78 [58]. The BNT-2 scores are not age-normed; we used total raw score (number correct) in our analyses.

#### Executive function

We administered two composite measures of EF shown to predict academic achievement in previous studies [59, 60].

The *Head-Toes-Knees-Shoulders* task (HTKS; [59]) is a three-part behavioral self-regulation measure of EF designed for use with children ages 4 to 8 years. The task involves incorporating rules and rule switches in order of increasing difficulty, in a “Simon Says” activity format. In Part 1, children are instructed to touch their head or their toes, but only when the examiner asks them to do the opposite; that is, when the examiner says, “touch your head,” children are expected to touch their toes, and vice versa. In Part 2, task demands increase because new commands are introduced that involve touching shoulders and knees. Once again, the child is instructed to do the opposite of what the examiner asks, and the child must remember to follow the rules from Part 1 in addition to the new commands. In Part 3, the new rules now involve switching between the previously introduced pairs of associated commands (e.g., touch your head now means touch your knees). For each part, children complete practice trials with corrective feedback followed by 10 test trials with no feedback. For each item, participants receive zero points for an incorrect response, one point for a self-corrected response, and two points for a correct response with no self-correcting. The HTKS has a reported internal reliability of α = 0.94 for children in their spring of kindergarten [59]. We used total scores out of 94, which included scores from 17 practice trials and 30 test trials.

The *Minnesota Executive Function Scale* (MEFS; [60]) is a tablet-based dimensional change card-sorting task designed for children ages 2 years through adulthood. Participants begin at one of seven levels based on age and are asked by an experimenter to electronically sort virtual cards into virtual boxes based on rules presented in order of increasing difficulty. Children in the present study began at level 5 (*n* = 126) or level 4 (*n* = 5) depending on their age at time of testing using the MEFS default settings, and moved forward or backward through the levels based on performance. Task demands increase across levels such that children need to switch between sorting rules, incorporate additional sorting rules, and change previously-learned sorting rules over the course of the task. The MEFS has a reported same-day test-retest reliability of α = 0.93 [61]. We reported participants’ standard scores, which were derived from total scores calculated with the MEFS algorithm, using both trial accuracy and response time [60], then standardized based on national norms (*M* = 100, *SD* = 15), and used total scores for subsequent analyses.

## Results

### Analytical strategy

Our aim was to assess mathematics as an outcome variable, with a specific focus on potential group differences in the cognitive profiles associated with mathematics. Our cognitive measures spanned MC_N_, verbal, EF, and numerical lexical ambiguity skills. All analyses were conducted in SPSS Version 24.

First, we completed preliminary descriptive statistics to confirm the fidelity of our matching procedures and to place our sample in the context of nationally normed scores. Second, we evaluated mathematics profile differences between girls with TS and the two matched peer groups using parallel sets of two-tailed paired sample *t*-tests of mathematics achievement, mathematics ability, MC_N_, and number word ambiguity interpretation skills. We also used ANOVA to evaluate the contributions of ratio bin and size control to the effects found in MC_N_ skills. Third, we examined profiles across our three groups of children, using paired sample *t*-tests to compare verbal and EF skills. In order to reduce variables in our analyses, we created composite scores by generating and then averaging sample-based z-scores, for two sets of scores. Specifically, we used raw scores from all 132 participants’ verbal subtest scores (KBIT-2 VK and BNT) to create a Verbal composite score, and their raw scores from two EF measures (MEFS and HTKS) to create an EF composite score. One child with TS had incomplete MEFS data due to an examiner oversight and therefore was excluded from primary analyses utilizing the EF composite score.

Our primary analyses of interest concerned relations across the domains. We closely examined Verbal and EF composite scores’ relation with mathematics outcomes, and MC_N_ and NAIT scores and their relation to mathematics outcomes. Noting contributions of MC_N_, Verbal composite, and EF composite scores to the variance in mathematics outcomes, we performed exploratory *post hoc* multiple regressions of these effects within group.

Our study design led to multiple statistical comparisons because of our focus on cognitive profiles comprised of skills in multiple domains, and because our comparison participants comprised two independent groups, each to be contrasted separately to participants with TS. Our reliance on paired *t*-tests exploits a more statistically powerful approach compared to unpaired *t*-tests. We also reduced the number of comparisons needed by using two separate composite scores as indicators of Verbal and EF skills. Still, to consider a maximally conservative interpretation of our data, we report Bonferroni corrections within each of the two parallel sets of multiple paired *t*-tests we reported. We also apply Bonferroni corrections of significance levels within group when considering correlations between mathematics outcomes and potential correlates.

### Descriptive statistics

We first examined the descriptive statistics for all study variables for which age referenced scores were available (summarized in Table 2). These scores showed that girls with TS scored, on average, at the 26^th^ percentile on the TEMA-3, at the 47^th^ percentile on the WJ-III AP, at the 64^th^ percentile on KBIT-2 VK, and at the 49^th^ percentile on the MEFS. This profile parallels earlier reports of the TS cognitive phenotype [e.g., 21], suggesting that our sample is representative of the TS population or, at least, of participants with TS from prior research. We examined differences in scores across girls with classic and mosaic TS karyotypes, and finding no significant effects attributable to karyotype differences we collapsed these subgroups in all subsequent analyses. Data from our comparison groups demonstrate that these children performed well within the average range on all four standard assessments, and thus are representative of the general population.

**Table 2:** Descriptive summary of mathematics, verbal, and EF standard scores for all 132 participants.

Measure	TS^a^	Verbal-Matched	MC_N_-Matched
TEMA-3 SS			
*M (SD)*	90.77 (12.76)	98.48 (13.58)	97.95 (14.07)
Range	65–120	68–130	68–125
WJ-III AP SS			
*M (SD)*	99.02 (10.83)	103.91 (10.16)	100.32 (12.70)
Range	74–122	82–121	67–124
KBIT-2 VK SS			
*M (SD)*	105.45 (10.83)	106.48 (12.23)	102.73 (16.37)
Range	75–125	65–125	65–135
MEFS SS			
*M (SD)*	99.86 (9.16)	100.84 (9.29)	100.68 (11.36)
Range	86–118	85–121	69–121

TS, Turner syndrome; MC_N_, nonsymbolic magnitude comparison; SS, standard score; TEMA-3, Test of Early Mathematics Ability–Third Edition; WJ-III AP, Woodcock Johnson Tests of Achievement–Third Edition, Applied Problems subtest; KBIT-2 VK, Kaufman Brief Intelligence Test–Second Edition, Verbal Knowledge subtest; MEFS, Minnesota Executive Function Scale.

^a^ The TS group has n = 43 participants with available MEFS SS.

We also used the descriptive statistics to confirm fidelity of our matching criteria, predicting no group differences on variables that served as match criteria. As predicted, there was no group difference between girls with TS and their Verbal-matched peers on the KBIT-2 VK standard score on which they were matched, *t*(43) = -1.19, *p* = .242. Moreover, there was no difference in overall Verbal composite scores, *t*(43) = 0.90, *p* = .373. Similarly, successful matching was indicated by a lack of significant differences in overall MC_N_ accuracy between girls with TS and their MC_N_-matched peers, *t*(43) = -1.47, *p* = .150. Compared to girls with TS, neither matched peer group differed significantly in age: *t*(43) = 0.58, *p* = .568 and *t*(43) = 1.86, *p* = .069, for the Verbal- and MC_N_-matched group; or months of schooling: *t*(43) = 0.82, *p* = .416 and *t*(43) = 0.37, *p* = .713, respectively.

Despite efforts to match pairs on as many criteria as possible, it was impossible to match pairs on all descriptive variables. As summarized in Table 1, parental education was similar across girls with TS and their Verbal-matched peers, *t*(39) = 0.07, *p* = .942, but girls with TS had parents with higher levels of education compared to their MC_N_-matched peers, *t*(38) = 2.31, *p* = .027. Compared to girls with TS, children in the Verbal-matched and MC_N_-matched comparison groups were more likely to be exposed to a language other than English in their home, *t*(42) = -2.15, *p* = .037, and *t*(40) = -5.11, *p* < .001, respectively, and were more likely to be eligible for free or reduced-price meals at school, *t*(40) = -2.72, *p* = .010 and *t*(40) = -4.61, *p* < .001, respectively. We return to the potential impacts of these differences in the discussion.

Based on our descriptive analyses, we paid particular attention to those children whose overall accuracy was below 75% on the Panamath task, in view of challenges reported by some researchers for similar MC_N_ tasks in young children. Although MC_N_ task results appear robust when administered with proper controls, even in young preschoolers [e.g., 31, 33, 34], our in-school administration differed from the typical laboratory settings in which these data from prior studies are reported, and we wanted to exclude children from profile analyses if their low accuracy was potentially invalid. However, low accuracy *per se* does not invalidate a child’s performance, so we examined item level and bin ratio level data for evidence that the child was performing either randomly or using a systematic erroneous alternative strategy (e.g., always choosing blue) for ten or more consecutive trials within the 88 trials. When examiner notes indicated concern about testing, we reviewed video tapes of testing sessions. We did not exclude data from children who were clearly engaged in the task, viewing stimuli before responding. We excluded data for children who consistently selected which set had “more” before seeing the two sets of dots, or made comments indicative of an alternative strategy (e.g., said “yellow always had more”). Based on these criteria, we excluded data from 12 of our 132 participants in any analyses involving MC_N_ scores. These participants were 5 girls with TS (4 in prekindergarten and 1 in Grade 1), 4 MC_N_-matched peers (3 in prekindergarten and 1 in kindergarten), and 3 were Verbal-matched peers (3 in prekindergarten and 1in kindergarten). Importantly, we found evidence of MC_N_ matching with *t*(37) = -1.35, *p* = .186 and without *t*(43) = -1.47, *p* = .150 these exclusions.

Similarly, and consistent with previous analysis of the NAIT task [25], participants were excluded from LNWB analyses if they never made an “I don’t know” response on the warm-up trials designed to train children that responding “I don’t know” was correct and expected for some items on the task when a quantity is unknown, or if they showed a preference for one of two responses on binary forced choice items by making the same choice on 75% or more force-choice trials. Based on these criteria, data was excluded from 14 participants of our 132 participants in any analyses involving NAIT LNWB. These participants were 6 girls with TS (4 in prekindergarten, 1 in kindergarten, and 1 in Grade 1), 3 Verbal-matched peers (2 in prekindergarten and 1 in kindergarten), and 5 MC_N_-matched peers (3 in prekindergarten and 2 in Grade 1).

### Primary analyses

In contrast with the standard assessments used for descriptive purposes, most of our experimental measures yielded only raw scores. For uniformity across the primary analyses that follow, we relied on raw scores from all assessments in the primary analyses and also to generate z-scores that were averaged to create the Verbal and EF composite scores. We did not combine the TEMA-3, WJ-III AP, and MC_N_ scores because our aim was to examine patterns of variance and covariance across these performance indices related to mathematical thinking and achievement. Likewise, as an exploratory construct, we examined NAIT LNWB errors individually, examining differences between groups and the relation between LNWB errors and either mathematics ability and achievement. Results from paired *t*-tests are summarized in Table 3, and correlations are summarized in Table 4. Bonferroni corrections result in a significance at the *p* < .008 level in the *t*-tests and *p* < .004 level in the correlational analyses.

**Table 3:** Group means and standard deviations for raw scores for all valid cases.

Measure	TS^a^	Verbal-Matched^b^	MC_N_-Matched^c^
TEMA-3 RS			
*M (SD)*	34.70 (17.79)	39.89 (18.08)**	38.05 (15.66)
Range	2–72	2–72	14–69
WJ-III AP RS			
*M (SD)*	21.82 (6.66)	23.23 (7.49)	21.68 (5.62)
Range	10–40	4–39	11–31
MC_N_ % Correct			
*M (SD)*	81.15 (12.20)	89. 22 (7.80)**	81.34 (11.28)
Range	52.27–97.73	62.50–98.86	55.68–97.73
NAIT LNWB			
*M (SD)*	13.89 (4.58)	12.45 (4.76)	13.18 (4.45)
Range	1–21	0–18	1–19
Verbal Composite			
*M (SD)*	0.12 (.87)	0.06 (0.90)	-0.18 (1.06)
Range	-2.00–1.64	-1.90–1.53	-2.38–1.91
KBIT-2 VK RS			
*M (SD)*	21.66 (6.64)	22.09 (6.68)	20.75 (7.91)
Range	7–37	8–38	5–39
BNT-2 RS			
*M (SD)*	33.84 (8.82)	32.18 (9.24)	29.39 (10.87)
Range	10–48	12–50	8–51
EF Composite			
*M (SD)*	-0.10 (0.85)	0.07 (0.89)	0.03 (0.88)
Range	-1.85–1.24	-1.89–1.33	-2.54–1.35
HTKS RS			
*M (SD)*	62.18 (22.04)	67.86 (27.20)	66.52 (21.66)
Range	4–89	0–93	2–92
MEFS RS			
*M (SD)*	62.51 (17.58)	64.30 (15.71)	63.75 (19.89)
Range	33–92	32–93	15–93

TS, Turner syndrome; RS, raw score; TEMA-3, Test of Early Mathematics Ability–Third Edition; WJ-III AP, Woodcock Johnson Tests of Achievement–Third Edition, Applied Problems subtest; MC_N_, nonsymbolic magnitude comparison; NAIT LNWB, Numerical Ambiguity Interpretation Task Total Large Number Word Bias Errors; Verbal Composite, based on KBIT-2 VK and BNT-2; KBIT-2 VK, Kaufman Brief Intelligence Test–Second Edition, Verbal Knowledge subtest; BNT-2, Boston Naming Test–Second Edition; EF Composite, based on HTKS and MEFS; HTKS, Head, Toes, Knees, Shoulders task; MEFS, Minnesota Executive Function Scale.

^a^Sample size in the TS group was 44 participants, except for MC_N_ percent correct (*n* = 39), NAIT LNWB (*n* = 38), and MEFS RS and EF Composite (*n* = 43).

^b^Sample size in the Verbal-matched group was 44 participants, except for MC_N_ percent correct (*n* = 41) and NAIT LNWB (*n* = 40).

^c^Sample size in the MC_N_-matched group was 44 participants, except for MC_N_ percent correct (*n* = 40) and NAIT LNWB (*n* = 39).

Rows in gray represent raw scores not subject to analysis because they were represented as part of a composite score.

** Significantly different from TS group, *p* < .008.

**Table 4:** Pairwise partial correlations between mathematics outcomes and primary correlates of interest, by group, controlling for age.

Measure	TS			Verbal-Matched			MC_N_-Matched		
	TEMA-3	WJ-III AP	MC_N_	TEMA-3	WJ-III AP	MC_N_	TEMA-3	WJ-III AP	MC_N_
MC_N_	.20	.28	-	.29	.38*	-	.56**	.48**	-
	*n* = 39	*n* = 39		*n* = 41	*n* = 41		*n* = 40	*n* = 40	
NAIT LNWB	-.22	-.09	-.06	-.29	-.31	.16	.02	-.20	-.13
	*n* = 38	*n* = 38	*n* = 35	*n* = 40	*n* = 40	*n* = 38	*n* = 39	*n* = 39	*n* = 36
Verbal Composite^a^	.28	.42*	.18	.32*	.49**	.01	.37*	.49**	.37*
	*n* = 44	*n* = 44	*n* = 39	*n* = 44	*n* = 44	*n* = 41	*n* = 44	*n* = 44	*n* = 40
EF Composite^a^	.17	.29	.35*	.52**	.53**	.26	.45**	.48**	.54**
	*n* = 43	*n* = 43	*n* = 38	*n* = 44	*n* = 44	*n* = 41	*n* = 44	*n* = 44	*n* = 40

TS, Turner syndrome; TEMA-3, Test of Early Mathematics Ability–Third Edition; WJ-III AP, Woodcock Johnson Tests of Achievement–Third Edition, Applied Problems subtest; MC_N_, nonsymbolic magnitude comparison; NAIT LNWB, Numerical Ambiguity Interpretation Task Total Large Number Word Bias Errors.

^a^ Verbal Composite score based on the Kaufman Brief Intelligence Test–Second Edition Verbal Knowledge subtest and the Boston Naming Test–Second Edition; EF Composite score based on the Head, Toes, Knees, Shoulders task and the Minnesota Executive Function Scale.

Statistically significant values appear in bold type face.

*p < .05

**p < .004, significant when applying Bonferroni correction within group.

#### Mathematics scores across groups

We examined four mathematics-related scores across participant groups, as two sets of paired *t*-tests. On the TEMA-3, girls with TS scored about 5 raw points lower than their Verbal-matched peers on this measure of mathematics ability, *t*(43) = -2.84, *p* = .007; this comparison remained significant with the Bonferroni correction. Girls with TS scored about 3 raw score points lower on the TEMA-3 than their MC_N_-matched peers, which was not significant, *t*(43) = -1.56, *p* = .125. On WJ-III AP, girls with TS scored only 1 raw score point lower compared to their Verbal-matched peers, *t*(43) = -1.97, *p* = .055, and had similarly comparable scores compared to their MC_N_-matched peers, *t*(43) = 0.17, *p* = .870.

**Fig 1:**
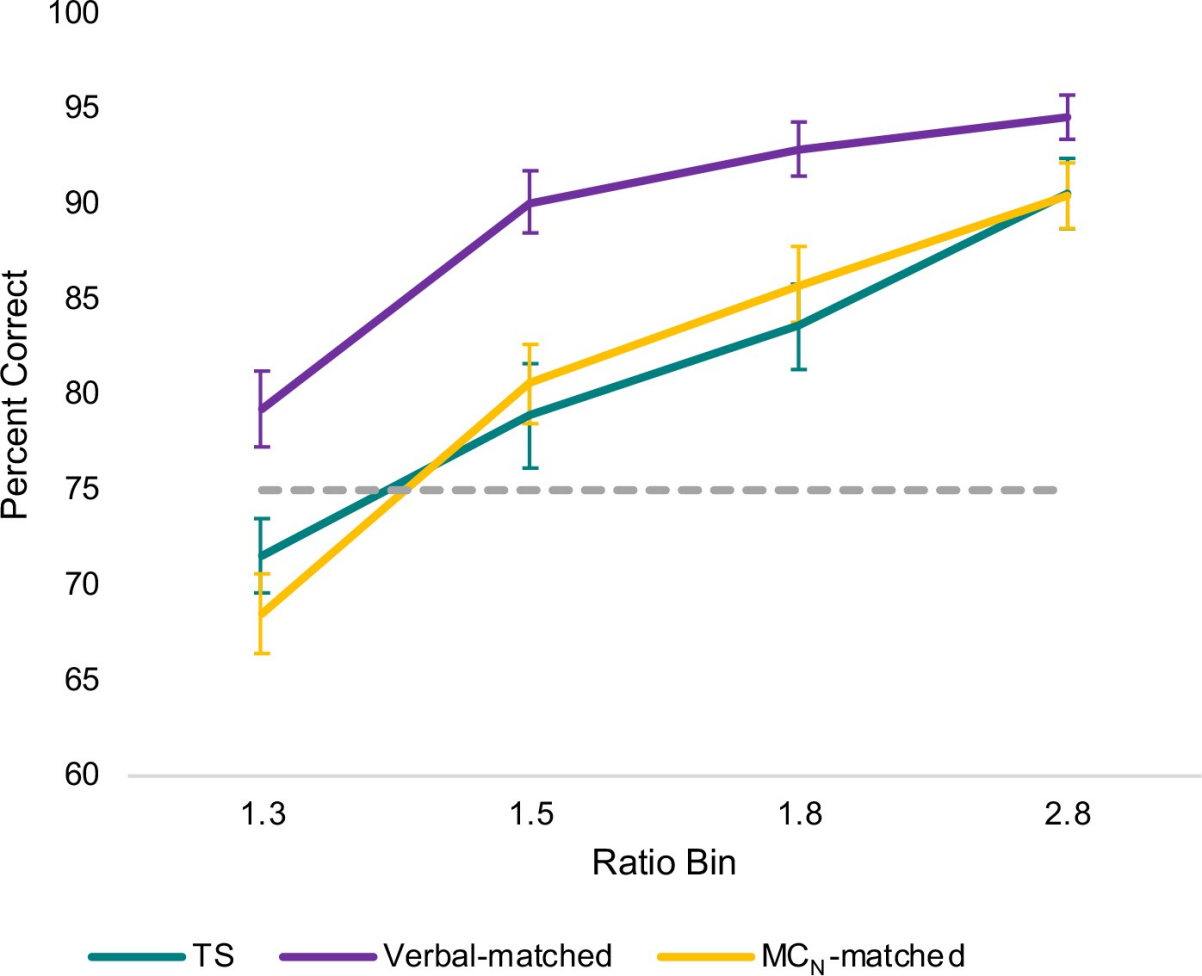
MC_N_ accuracy across ratio bin. Relation between ratio bin (ratio of the dot arrays) and overall percent correct on the MC_N_ task for each group. The threshold for reliably accurate magnitude comparison, which we define as accuracy of 75% or above, is marked by the dotted line. Error bars represent standard error of the mean. Data include *n* = 39 children with TS, *n* = 41 Verbal-matched children, and *n* = 40 MC_N_-matched children.

**Fig 2:**
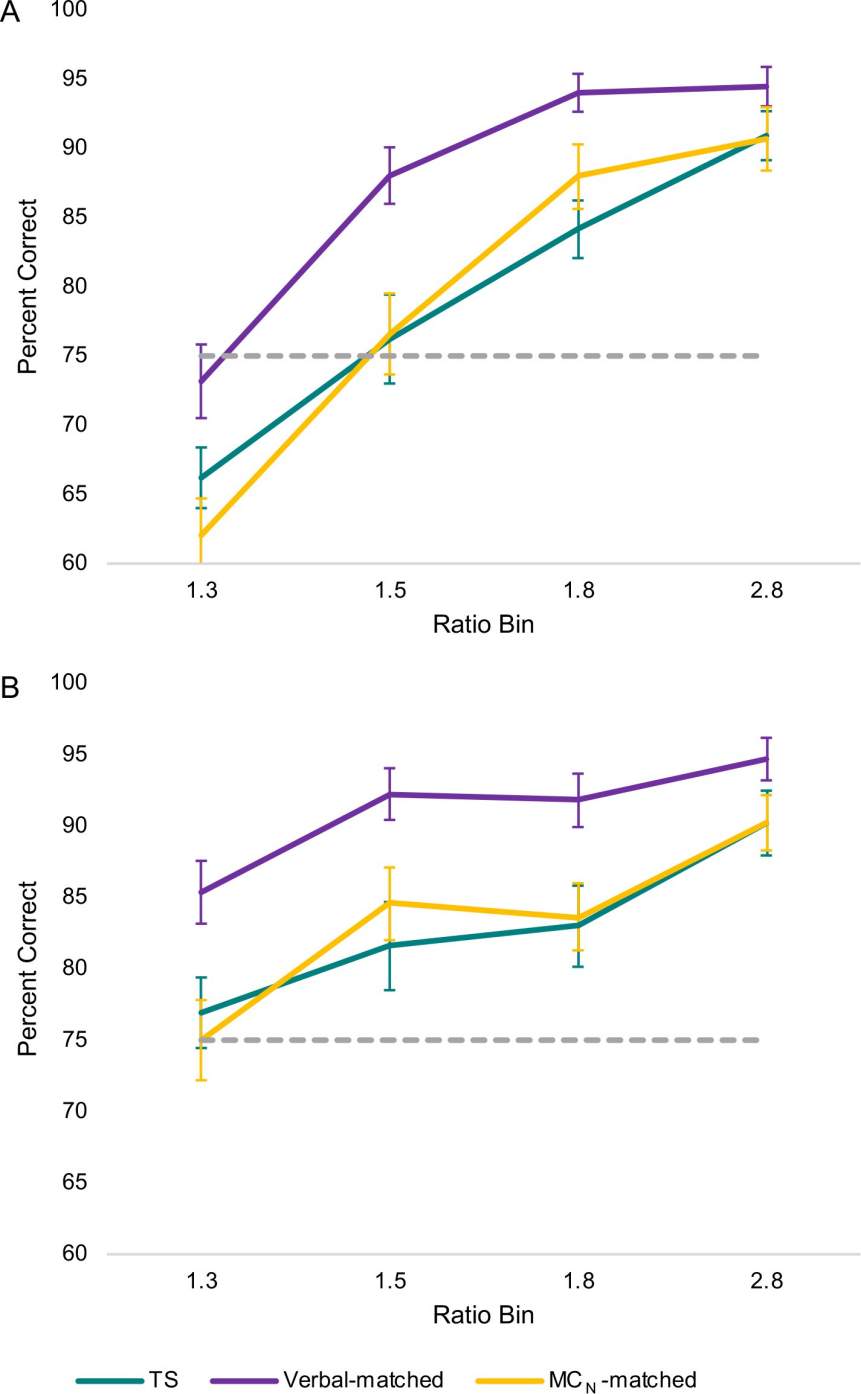
Mean MC_N_ accuracy by size control, across ratio bin (Ratio of dot arrays). Panel A shows accuracy (percent correct) when dots were size-controlled (the more numerous and less numerous sides had the same total number of pixels). Panel B shows percent correct when dots were not size-controlled (the more numerous side had more dots and more total pixels than the less numerous side). Reliably accurate magnitude comparison, which we define as accuracy at or above the 75% threshold, is marked by the dotted line. Error bars represent standard error of the mean. Data include *n* = 39 children with TS, *n* = 41 Verbal-matched children, and *n* = 40 MC_N_-matched children.

A key contribution of the current study was our assessment of MC_N_ skills of girls with TS. As summarized in Figs 1 and 2, accuracy (percent correct) on the MC_N_ task differed between girls with TS and their Verbal-matched peers, across each ratio bin of the dot arrays, regardless of whether the dot displays were size-controlled.

A paired *t*-test revealed a significant difference between girls with TS and their Verbal-matched peers in overall MC_N_ accuracy, *t*(36) = -4.34, *p* < .001; a difference that remained significant when applying a Bonferroni correction. However, the *t*-test did not account for ratio bin or size control, which we therefore assessed using a 2 (Group) × 4 (Ratio bin) × 2 (Size control) ANOVA. Due to violations of sphericity assumptions across ratio bins, we used Huynh-Feldt corrected tests of relevant effects. Comparing girls with TS to their Verbal-matched peers, there were main effects of Group, *F*(1, 36) = 18.82, *p* < .001; Ratio bin, *F*(2.13, 76.53) = 73.92, *p* < .001; and Size control, *F*(1, 36) = 17.97, *p* < .001. We observed a two-way interaction of Ratio bin × Size control, *F*(2.55, 91.88) = 11.53, *p* < .001, and Ratio bin × Group, *F*(2.46, 88.53) = 3.64, *p* = .022, but not Group × Size control, *F*(1, 36) = 0.09, *p* = .769 (Fig 2). There was no three-way interaction, *F*(2.75, 99.07) = 0.42, *p* = .723. Bonferroni-corrected, *post hoc* follow-up of the Group × Ratio bin interaction revealed significant differences between girls with TS and their Verbal-matched peers in overall accuracy at a ratio bin of 1.3, *t*(36) = -3.37, *p* = .002, a ratio bin of 1.5, *t*(36) = -4.19, *p* < .001, and a ratio bin of 1.8, *t*(36) = -4.31, *p* < .001; the difference at a ratio bin of 2.8, *t*(36) = -2.23, *p* = .032, did not meet significance criteria correcting for *post hoc* multiple comparisons.

As expected, based on the matching criteria as reported in preliminary results, girls with TS did not differ from their MC_N_-matched peers on overall MC_N_ accuracy. Still, paired matching on *overall* accuracy did not guarantee that no differences would emerge relative to ratio bin or size control, so we similarly examined these using a 2 (Group) × 4 (Ratio bin) × 2 (Size control) ANOVA. Again, due to violations of sphericity assumptions across ratio bins, we used Huynh-Feldt corrected tests of relevant effects. We found no main effect of Group, *F*(1, 37) = 1.82, *p* = 186. We observed main effects of Ratio bin, *F*(2.50, 92.48) = 108.87, *p* < .001, and of Size control, *F*(1, 37) = 12.57, *p* = .001. There was a Ratio bin × Size control interaction, *F*(2.52, 93.15) = 12.06, *p* < .001 (Fig 2). There was no significant Group × Ratio bin *F*(2.50, 92.63) = 2.72, *p* = .059 or Group × Size control interaction, *F*(1, 37) = 0.00, *p* = 1.00, nor was there a three-way interaction *F*(2.65, 97.91) = 0.43, *p* = .704.

Finally, we considered LNWB errors on the NAIT as a potential predictor of variation in early childhood mathematics that may differ between girls with TS and children that share aspects of the TS cognitive profile. There was no significant difference between girls with TS and the Verbal-matched group, *t*(36) = 1.45, *p* = .157; nor the MC_N_-matched group, *t*(33) = 0.72, *p* = .478.

#### Correlations among mathematics scores

We next considered the correlation between MC_N_, NAIT LNWB errors, and broad mathematics outcomes in ability and achievement, separately for each of our three participant groups (Table 4). We relied on raw scores for all measures controlling for age. Within each group, TEMA-3 and WJ-III AP correlated with each other, *r* = 0.70 in the TS group, *r* = 0.77 in Verbal-matched peers, and *r* = 0.78 in MC_N_-matched peers (all *p*s < .001); but their relation to MC_N_ accuracy differed across groups. First, among girls with TS, TEMA-3 and WJ-III AP were not significantly correlated with overall MC_N_ accuracy when controlling for age (*p* = .235 and *p* = .085, respectively). By contrast, among girls in the Verbal-matched comparison group, WJ-III AP was moderately and significantly correlated with MC_N_ accuracy (*p* = .016), but not TEMA-3 (*p* = .075). Among girls in the MC_N_-matched group, TEMA-3 and WJ-III AP were correlated with MC_N_ accuracy (*p* < .001 and *p* = .002, respectively). Applying a Bonferroni correction within group to these correlations, only the correlations in the MC_N_-matched peer group remain significant. In contrast, NAIT LNWB showed no significant effects in any group with any measure of mathematics when controlling for age (all *p*s > .056).

#### Verbal and EF skills: Group differences and correlations with mathematics

As reported in the preliminary analyses, girls with TS and their Verbal-matched peers had KBIT-2 VK standard scores at approximately the 64^th^ percentile. There were also no significant differences in Verbal composite scores between girls with TS and their Verbal-matched peers, *t*(43) = 0.90, *p* = .373. Girls with TS also did not differ in Verbal composite score compared to their MC_N_-matched peers, *t*(43) = 1.78, *p* = .082. Similarly, MEFS standard scores were close to 100 for all three participant groups, and paired *t*-tests to assess composite EF scores showed no significant difference between TS and Verbal-matched peers, *t*(42) = -1.05, *p* = .298, nor differences between TS and MC_N_-matched peers, *t*(42) = -0.72, *p* = .476.

Beginning with the Verbal composite scores, we examined the correlation between our two composite scores, alongside MC_N_ and broad mathematics outcomes separately, for each of our three groups of participants (Table 4). Among girls with TS, Verbal composite scores correlated significantly with WJ-III AP (*p* = .005), but not with TEMA-3 (*p* = .073) nor MC_N_ (*p* = .294) scores. The correlation with WJ-III AP did not reach significance criteria controlling for multiple correlations. We observed a similar pattern in the Verbal-matched comparison group, wherein the Verbal composite score was correlated with WJ-III AP (*p* = .001), but not with TEMA-3 (*p* = .036), nor with MC_N_ (*p* = .952) scores, when controlling for multiple correlations. Among girls in the MC_N_-matched group, the Verbal composite score was moderately correlated with all three mathematics outcomes, including with the WJ-III AP (*p* = .001), TEMA-3 (*p* = .015), and MC_N_ accuracy (*p =* .021) scores, but only the correlation with WJ-III AP was significant when controlling for multiple correlations.

Turning to the correlations between the EF composite score and broad mathematics outcomes, we saw different patterns across the three participant groups (Table 4). Among girls with TS, the EF composite was not significantly correlated with TEMA-3 (*p* = .276) or WJ-III AP (*p* = .067), and the correlation with MC_N_ accuracy (*p* = .034) did not meet significance when correcting for multiple comparisons. Among girls in the Verbal-matched comparison group, the EF composite score was significantly correlated with TEMA-3 (p < .001) and WJ-III AP (p < .001) when correcting for multiple comparisons. The correlation between the EF composite score and MC_N_ accuracy (*p* = .105) was not significant. Finally, among girls in the MC_N_-matched group, the EF composite score was significantly correlated with TEMA-3 (*p* = .002) and WJ-III AP (*p* = .001), and there was a significant correlation between EF composite and MC_N_ accuracy (*p* < .001), when correcting for multiple comparisons,

#### *Post hoc* analyses: MC_N_ accuracy, EF, and mathematics

Among girls with TS or their matched peers, MC_N_ accuracy, Verbal composite, and EF composite scores emerged as skills significantly correlated with mathematical thinking, to different degrees across all three groups. We therefore explored these effects further with *post hoc* multiple regressions. Aligned with our previous analyses, age was entered into the model, because we expected developmental growth in mathematics ability and achievement raw scores and therefore controlled for it in this analysis of raw scores. MC_N_ accuracy, Verbal composite, and EF composite scores were simultaneously entered into the model predicting TEMA-3 (Table 5) and WJ-III AP (Table 6). These results differed across groups and measure of mathematics outcome.

**Table 5:** Multiple regression predicting TEMA-3 scores.

	TS			Verbal-Matched			MC_N_-Matched		
	(*n* = 38)			(*n* = 41)			(*n* = 40)		
	β	*t*	*p*	β	*t*	*p*	β	*t*	*P*
Age	**.66**	**5.01**	**< .001**	**.60**	**5.54**	**< .001**	**.48**	**4.34**	**< .001**
MC_N_	.06	0.64	.529	.11	1.19	.242	**.30**	**2.54**	**.016**
Verbal Composite^a^	.17	1.38	.177	.06	0.61	.545	.07	0.53	.603
EF Composite^a^	.09	0.79	.436	**.26**	**2.56**	**.015**	.17	1.19	.242

TEMA-3, Test of Early Mathematics Ability–Third Edition; TS, Turner syndrome; MC_N_, nonsymbolic magnitude comparison.

^a^ Verbal Composite score is based on the Kaufman Brief Intelligence Test–Second Edition Verbal Knowledge subtest and the Boston Naming Test–Second Edition; EF Composite score is based on the Head, Toes, Knees, Shoulders task and the Minnesota Executive Function Scale.

Statistically significant values appear in bold type face.

**Table 6:** Multiple regression predicting WJ-III AP raw scores.

	TS			Verbal-Matched			MC_N_-Matched		
	(*n* = 38)			(*n* = 41)			(*n* = 40)		
	β	*T*	*P*	β	*t*	*p*	β	*t*	*p*
Age	**.42**	**3.15**	**.003**	**.50**	**5.15**	**< .001**	**.38**	**3.17**	**.003**
MC_N_	.08	0.76	.455	**.19**	**2.26**	**.030**	.22	1.73	.092
Verbal Composite^a^	**.34**	**2.82**	**.008**	**.21**	**2.26**	**.030**	.27	1.87	.069
EF Composite^a^	.20	1.79	.084	.18	1.98	.055	.14	0.87	.390

WJ-III AP, Woodcock Johnson Tests of Achievement–Third Edition, Applied Problems subtest; TS, Turner syndrome; MC_N_, nonsymbolic magnitude comparison

^a^ Verbal Composite score is based on the Kaufman Brief Intelligence Test–Second Edition Verbal Knowledge subtest and the Boston Naming Test–Second Edition; EF Composite score is based on the Head, Toes, Knees, Shoulders task and the Minnesota Executive Function Scale.

Statistically significant values appear in bold type face.

Among children with TS, only age emerged as an independent predictor of TEMA-3 raw scores (*p* < .001). This contrasts with the Verbal-matched group in which age (p < .001) and EF Composite (*p* = .015) acted as independent predictors of concurrent TEMA-3 scores and the MC_N_-matched group in which age (p < .001) and MC_N_ accuracy (*p* = .016) acted as independent predictors of TEMA-3. Considering WJ-III raw scores (Table 6), in the TS group age (*p* = .003) and Verbal composite (*p* = .008) were significant predictors. This effect of age (*p* < .001) and Verbal composite (*p* = .030) was also seen in the Verbal-matched group with an additional independent effect of MC_N_ accuracy (*p* = .030). In the MC_N_-matched group, only age (*p* = .003) emerged as a predictor of WJ-III AP raw scores.

## Discussion

We carried out the current study to examine whether the cognitive phenotype of girls with TS manifests with mathematics difficulties in the early school years, a time period during which early identification can offer significant benefits associated with early prevention or intervention. Guided by earlier work on the TS cognitive profile and the foundational role of early numerical skills for mathematics achievement in general, we included measures of informal and formal mathematics ability (i.e., TEMA-3) and an applied mathematics achievement measure (i.e., WJ-III AP) as outcomes of interest among our three participant groups. We were specifically interested in how cognitive profiles and their relation to mathematics outcomes differentiate children with or without TS, and the implication of these profiles for understanding variation in emerging mathematics skills in general. Towards these goals, we included measures of MC_N_, verbal, and EF skills in our assessment battery, and explored the potential role numerical lexical ambiguity may play in elucidating the confluence of numerical and verbal skills in TS. We included a novel combination of peer comparison groups in the study in order to evaluate the specificity with which each of these skills is associated with mathematics outcomes in children with TS, compared to children without TS who share a single feature of the presumed TS profile (i.e., relatively high verbal or low MC_N_ skills). As outlined in the following sections, although only some of our hypotheses were supported, our findings contribute to our understanding of the TS phenotype and to cognitive correlates of early mathematics.

### Mathematics scores

#### Mathematics outcomes

Based on prior research, we anticipated low scores on mathematics ability and mathematics achievement in girls with TS. On average, our participants with TS had lower scores on a measure of mathematics ability (TEMA-3), relative to population standardized scores (Table 2); and significantly lower TEMA-3 raw scores than their Verbal-matched peers, consistent with the broader literature on the TS phenotype (Table 3). Girls with TS had lower TEMA-3 scores compared to their peers individually matched on MC_N_ accuracy, but this difference did not reach significance. On a measure of mathematics achievement (WJ-III AP), girls with TS had scores similar to their Verbal- and MC_N_-matched peers, with all three groups scoring well within the average range relative to population standardized scores, and the TS group scoring at the 47^th^ percentile on average. This pattern of lower informal mathematics ability and higher mathematics achievement is consistent with earlier reports of average achievement despite poor numerical skills in girls with TS (e.g., [21]); however, those earlier reports concerned the performance of older girls with TS, including those in late elementary to middle school. Another study of numerical and mathematics achievement skills in young children with TS (from Grades K to 3) did *not* report lower performance on specific individual TEMA items but did report a heightened risk for mathematics learning difficulties over time [11]). If mathematics difficulties in TS manifest on achievement tests rather than tests like the TEMA-3 that focus on informal numerical abilities, this manifestation may not be evident until later in the school age years. Our inclusion of prekindergartners may explain our null findings in mathematics achievement levels– 25% of our participants were in prekindergarten and therefore had minimal school mathematics instruction, potentially limiting the extent to which our measure of mathematics achievement differentiated children with or without TS. It is noteworthy that, among children with TS, cognitive correlates of math emerged on the achievement test (WJ-III AP) but not the TEMA-3 (Tables 5 and 6).

Nevertheless, lower scores in the TS group on the TEMA-3, a measure with heavy emphasis on informal numerical skills, bolster previous reports of early mathematics difficulties in girls with TS from kindergarten to third grade, and extend these prior findings to another sample of girls with TS that included 4-year-old prekindergartners. Recognizing that the TS early mathematics profile emerges prior to formal schooling is important, because it informs recommendations to promote early mathematical thinking at home and in preschool prior to kindergarten entry [62]. Accordingly, our findings support the clinical practice guidelines for care of children and adults with TS [63] that recommend “assessments at key transitional stages in schooling” (p. G43). Our findings implicate that this recommendation is relevant to mathematics specifically, at or before school entry. We note that recommendations to promote early mathematics skills at home (i.e., prior to starting school) also apply to the general population [64] and focus on mathematics integrated into play and daily routines [65], since these are related to later mathematics outcomes [66]. Such resources are, therefore, likely to be applicable also to children with TS.

#### Magnitude comparison skills

Although not a traditional early mathematics task per se, the MC_N_ task was relevant to our study as both a developmentally-appropriate basic numerical processing indicator in early childhood and a potential domain-specific correlate of mathematics outcomes. In our study, girls with TS were less accurate on the MC_N_ task relative to their Verbal-matched peers, and this difference held when correcting for multiple comparisons (Table 3). (Obviously, there was no group difference between the group with TS and their MC_N_-matched peers.) To our knowledge, our study is the first to show that young girls with TS, as a group, have lower than expected accuracy on an MC_N_ task. Moreover, this lower MC_N_ accuracy in girls with TS compared to their Verbal-matched peers emerged regardless of size-control of the displays, and despite the fact that all trials involved simultaneous (rather than sequential) displays of numerosities. This finding suggests that the visuo-spatial or inhibitory and working memory aspects of the displays could not have been the sole mechanisms underlying our findings, a noteworthy consideration because these factors have been identified by others as relevant to understanding individual differences in MC_N_ skills in general (e.g., [67]) and in older children and adults with TS [35]. The group differences in MC_N_ scores were not likely to be an artifact of a higher incidence of invalid scores in the TS group, because we explicitly excluded MC_N_ data from all 12 participants whose MC_N_ performance was potentially invalid. The exclusion of these potentially invalid scores was based on conservative criteria, resulting largely from our youngest participants enrolled in prekindergarten, and occurred across all three participant groups.

Among all cases that met strict criteria for validity, we were interested in whether the MC_N_ and TEMA-3 correlations reported in prior studies of individuals *without* TS would replicate in our TS sample. Among both matched peer participant groups in our study, we found that MC_N_ and TEMA-3 partial correlations (.29 or .56 in our verbal- or MC_N_-matched peers, respectively) were similar to effect sizes reported among other samples of young children ~ .28 [31] to .52 [33]. In contrast, the partial correlation between MC_N_ and the TEMA-3 observed in girls with TS (.20) did not meet our significance threshold, and a smaller effect size compared to the of MC_N_ and TEMA-3 correlations reported in other studies of mathematics skills in early childhood (e.g., [31, 33, 68, 69]). On the one hand, these findings may suggest that there is nothing unique about the MC_N_ and TEMA-3 correlation in TS, because a correlation of .20 approaches those earlier reported levels; on the other hand, these correlations among girls with TS were weaker relative to the other groups, did not reach statistical significance when correcting for multiple comparisons, and thus may be suggestive of a disparate trend in the TS group compared to peers.

When focusing on formal mathematics achievement rather than performance on the TEMA-3, partial correlations between MC_N_ and the WJ-III AP achievement test (controlling for age) exceeded expected effect size levels, across both peer groups and in the group with TS (.28) although the latter was not significant. Moreover, MC_N_ accuracy did not act as an independent predictor of variance in TEMA-3 nor WJ-III scores in the TS group; this may suggest that MC_N_ accuracy may be unrelated to variance in emerging math abilities or achievement in the TS phenotype.

To summarize the results of mathematics outcomes, the most robust finding was that of lower MC_N_ skills in girls with TS compared to peers with similar verbal skills, and that girls with TS and their MC_N_-matched peers had comparable scores on mathematics ability and achievement measures. At first glance–and based on our limited data–we may interpret the latter similarities as suggesting that girls with TS have a mathematical profile similar to that of their MC_N_-matched peers, such that there is no unique TS mathematics profile. Upon closer scrutiny, however, this interpretation conflicts with the differing patterns of correlations observed between these two groups of participants, because accuracy on the MC_N_ task was *not* significantly correlated with TEMA-3 scores among girls with TS but the correlation was strong and significant in the MC_N_-matched group. The significant relation between mathematics ability and MC_N_ accuracy found in the MC_N_-matched group is consistent with a meta-analysis of MC_N_ and mathematics [70]; performance by the TS group is not. Moreover, the lack of a significant correlation between MC_N_ and mathematics scores in the TS group was not simply the result of a restricted range of scores in the TS group, as TEMA-3 and WJ-III AP standard scores in this group spanned from 65–118 and 74–121, respectively. Although these correlations are insufficient for clarifying the nuances of mathematics ability in young girls with TS, the findings support the notion of a unique TS mathematics profile with respect to numerical processing skills and their role in early mathematics achievement; these profiles were examined further with the inclusion of domain-general correlates of mathematics.

### Inconsistent evidence for verbal strength and EF differences

Correlations between verbal or EF measures and mathematics may reveal unique patterns between girls with TS and their peers. Our focus on verbal skills stemmed from prior evidence suggesting the potential for verbal skills strengths in the TS group [15] and the hypothesis that verbal compensatory processes contribute to mathematics achievement specifically. We included EF measures in our study because of the importance of EF skills in early mathematics outcomes in the general population [e.g., 23, 58], and prior evidence of variable EF skills in the TS phenotype [24]. We therefore evaluated differences in skill level between groups, and associations within groups, for further insights. These findings supported only some of our original hypotheses.

Although we did find some evidence of a relative verbal strength *within* the TS group (e.g., KBIT-2 VK standard score at the 64^th^ percentile compared to TEMA-3 standard score at the 26^th^ percentile), comparison *between* groups showed no statistically significant differences when considering a composite verbal score, unlike other findings that have suggested verbal strength relative to peers [15]. Our lack of support for verbal strengths in the TS group compared to their MC_N_-matched peers was particularly striking when we consider that the MC_N_-matched comparison group was much more linguistically diverse in their home language exposure and both of our assessments of verbal skills were in English. That is, girls with TS did not have a consistent verbal skills advantage, even compared to children who had significantly less frequent English exposure in the home.

When we examined evidence of verbal compensatory skills contributing to mathematical achievement in girl with TS, findings were in the predicted direction and, in terms of effect size, suggested that verbal skills accounted for 17.5% of the variance in mathematics achievement (i.e., WJ-III AP scores; Table 4) in the TS group while controlling for age differences. Still, the relation, though moderate in size, did not reach significance when controlling for multiple correlations; moreover, correlations of this magnitude between the verbal composite and WJ-III AP scores were observed in all three groups. Complementing these findings, however, the exploratory regression analysis showed the Verbal composite score (but not MC_N_ accuracy or EF composite score) accounted for *unique* variance in mathematics achievement among girls with TS. Together, these findings provide some initial support for the hypothesis that verbal skills may contribute to variability in early childhood mathematics achievement in girls we TS in a manner not observed among their peers with similar low MC_N_ scores, and consistent with other children who have a modest verbal strength.

The weaker (and not significant) association between Verbal composite and the TEMA-3 was consistent with prior studies that found word identification was not associated with TEMA-3 in 5- and 6-year-old girls with TS, but was correlated in matched peers [20]. However, word identification skills are only weakly correlated with vocabulary in early childhood. This weaker correlation is inconsistent with our notion of a verbal compensatory mechanism supporting the mathematics skills of girls with TS. Alternatively, the results may indicate that, to the extent to which the girls with TS have a verbal advantage, they are not yet using these skills in the early school years to improve their mathematics performance, perhaps particularly on informal mathematics items which may not afford a verbal strategy. Early mathematics (prekindergarten to kindergarten) relies heavily on counting and number skills, at least in the U.S.; perhaps it is not until instruction engages children in mathematics operations and problem solving that the verbal compensatory skills play a significant role. In prior studies, we have shown that girls with TS make different types of arithmetic errors compared to their peers [43, 71], which may reflect differences in strategy selection when solving mathematics problems in middle childhood. It remains to be determined whether later deployment of verbal skills mediates these and earlier mathematical differences.

Our hypothesis concerning the role of verbal skills in TS also motivated us to include a task that involved interpreting numerically ambiguous lexical content (the NAIT), which we hypothesized would differentiate our participant groups and predict mathematics outcomes in the TS group specifically. We did not, however, find support for either of these hypotheses. The rate of responses consistent with a LNWB, which is a tendency to ignore relevant context in favor of the larger number word, was similar across all three groups, and in all groups the correlations between the LNWB and mathematics scores were not significant.

We would not expect any single cognitive measure to be a sole contributor to mathematical thinking and achievement, and thus also considered the contributions of EF skills to mathematics ability and MC_N_ accuracy, and whether EF skills may partially account for some of the differences we observed in how MC_N_ accuracy was associated with mathematics ability in girls with TS or their Verbal-matched peers–as discussed earlier in this section. Notably, we found no differences in mean level of performance on an EF composite score across groups, consistent with previous work suggesting that EF differences may not emerge until third grade when comparing girls with TS and their peers [21].

Still, correlational patterns differed when examining profiles between girls with or without TS. The EF composite score was not associated with mathematics ability or achievement in the TS group. In contrast, and consistent with the broader literature, EF was moderately associated with mathematics ability and achievement in both peer groups, as aforementioned, accounting for between 20% - 28% of the variance in mathematics outcomes when controlling for age. Across all groups, correlations of EF composite and MC_N_ accuracy ranging from .26 - .54 were consistent with others who have suggested a contribution of EF to differences in MC_N_ tasks [72], though in the present study these correlations were not significant when controlling for multiple correlations except in the MC_N_-matched group. Together, it does not appear that composite EF measures would account for mathematics differences in girls with TS during early childhood. Similar to verbal skills, open questions remain as to whether relations between mathematics and EF skills emerge later in development among girls with TS.

### Study limitations

Though the present study makes several novel contributions, there were several limitations that temper our conclusions, including the measures to which we limited, our specific comparison group composition, and our limited sample size. Like many studies, ours was constrained in scope in terms of age range and measures, although it is the first study of TS of its size, to our knowledge, that focuses exclusively on this age group.

Although we took care to choose measures relevant to the TS and wider mathematics literature, we were constrained to a finite testing battery appropriate for this age group and most relevant to the hypotheses we tested. It is possible that other tasks would reveal different results and may have provided more conclusive support for the role of EF skills. We did not, for instance, include sequential MC_N_ trials on our MC_N_ task like the one that Attout and colleagues used with their older participants with TS [35], so we cannot address whether increasing EF demands in an MC_N_ task would lead to even more pronounced MC_N_ differences in young girls with TS compared to their peers, nor comment more precisely on how spatial working memory contributes to our overall findings. These questions extend beyond our study aims to better understand the roles that MC_N_ and verbal skills play in the TS phenotype, and how MC_N_, verbal, and EF skill profiles differ between children with or without TS. We strongly believe that reliance on a more basic MC_N_ task is an appropriate starting point for understanding MC_N_ skills in young children with TS, and that it is likely that sequential MC_N_ trials presented to adolescents and adults in Attout et al.’s study [35] would be too challenging for the young children in our age group, as would be administering multiple versions of MC_N_ tasks in this age group. Still, we found group differences on *simultaneous* MC_N_ trials, whereas Attout and colleagues did not. This difference may reflect developmental differences in MC_N_ performance and differences in task demands across studies. We also found that group differences in MC_N_ accuracy were independent of spatial extent controls. Our findings contribute to the emerging picture of developmental differences in MC_N_ skills in girls with TS.

Generally, none of our covariate measures (verbal, EF, MC_N_, and number word ambiguity interpretation) correlated with mathematics *ability* in the TS group as measured by the TEMA-3 so we are unable to comment more directly on specific or general correlates of mathematics ability in the TS group. We found that patterns of correlations differed between groups, suggesting that interactions between cognitive skills and mathematics may operate uniquely in girls with TS. Still, our sample size precluded direct comparison of the correlations across groups, though we were able to comment on patterns of differences relative to null correlations.

TS occurs in 1 in 2000 to 1 in 2500 live female births, which generally necessitates nationwide sampling when evaluating cognitive outcome measures in a targeted age range. Thus, despite (to our knowledge) being the largest sample of participants in a study focused on early mathematics in TS to date, the study is underpowered for identifying small effect sizes. We also recognize that barriers to participation in a study like this may have contributed to a sample that was not nationally representative of girls with TS, and that differed from the comparison group that was not geographically diverse. Though we know that all children with TS in the study were in mainstream educational settings (i.e., attending school in regular classrooms), as this was a participation criterion for all three participant groups, we were not aware of which children across all participant groups had individualized education plans. Most of the participants in the comparison groups attended public school in the same greater urban region, whereas there was a wider range of schools represented among the group with TS attending schools across the U.S. This is a limitation of the study, but one that is difficult to avoid in the context of a nationwide recruitment of a special population. The groups also differed in their rate of free or reduced-price school meals eligibility (as a proxy for poverty) and in their exposure to languages other than English in their home (as a proxy for English Language Learner [ELL] status), which cannot be readily controlled for in our analyses. In the U.S., poverty and ELL status are associated with higher rates of mathematics underachievement (e.g., [73, 74]). Nevertheless, children with TS had lower mathematics scores than their Verbal-matched peers despite lacking these potential social disadvantages. Moreover, children with TS did *not* have a consistent verbal advantage, despite having more uniform exposure to English in the home. Therefore, differences in free or reduced-price meal status and ELL status are unlikely to underlie this set of results, but we are unable to rule out that these factors may have contributed to differences between groups.

Finally, our study was cross sectional and correlational, focusing on the relation between potential domain-specific and domain-general correlates of mathematics. Although the results are not causal, they do offer critical insights into potential pathways to, and alternative interventions for, girls with TS as avenues for further study.

### Conclusions

The current study contributes to our knowledge of the TS phenotype and of MLDs more generally. Like studies of the general population, the TS phenotype did not manifest as a homogenous MLD group, as expected. Heterogeneity emerged first within the range of mathematics skills, and later in the associations we explored between mathematics and other cognitive skills. That is, our findings *do* support the notion that TS increases a child’s likelihood of mathematics differences, and yet many participants with TS had age appropriate mathematics scores. This combination demonstrates the variation in manifestation of TS reported in other studies of TS. Mathematics differences, if present in early childhood, may be subtle in young children with TS, consistent with earlier findings that specificity of mathematics difficulties does not emerge early in development in TS [11].

In terms of the cognitive associations with mathematics scores, we did not identify any clear pathway to mathematics achievement for girls with TS, but did note important group differences in the relation between their mathematics achievement and the cognitive skills we examined. Our findings point to MC_N_ underperformance in TS, but this was, surprisingly, *unrelated* to the mathematics outcomes of the girls with TS in our study. Perhaps the MC_N_ task measures different skills in different children–particularly if there are individual or developmental variations in children’s approaches to engage in MC_N_ tasks–which may contribute to inconsistent findings across studies examining these correlations. Alternatively, differential correlational patterns may hint at different approaches children take to engaging mathematically on problems such as those included on the TEMA-3 and WJ-III AP tasks, including any reliance (partial or otherwise) on MC_N_ skills, and that relative reliance on specific cognitive skills may vary across the early school years as specific instructional expectations vary from prekindergarten to Grade 3. Taking this notion further, reliance on different cognitive skills across groups may also reflect within-group variation among girls with TS, for whom the degree of engagement of verbal or EF skills may depend, for instance, level of MC_N_ skills. Our sample size prevented exploration of these differences.

Although we were unable to reveal the full story of how children with TS approach mathematics during the early school years, the current results partially supported our hypothesis that girls with TS may rely on verbal skills to achieve in mathematics, albeit based only on associations that emerged. These associations point to future avenues of productive research. As discussed, young children with TS have some verbal advantage relative to their own mathematics skills, but we see only partial evidence of verbal skills’ contribution to variation in mathematics achievement at a young age, and less evidence of a contribution to numerical skills captured by the TEMA-3. It remains an open question whether and how verbal skills might be harnessed to improve mathematics outcomes, for children with TS and those with a similar constellation of skills; whether efforts to support EF skills offer any particular advantage; or whether verbal and EF contributions to mathematics vary across development in TS in a way that differs from how verbal and EF skills influence the development of mathematics skills in the general population.

Importantly, in the present study we identified three group-level profiles that revealed nuances in the relation between mathematics outcomes and cognitive skills. Children that share key aspects of the TS phenotype (verbal skills or basic numerical processing) did have predicted differences and similarities in mathematics outcomes. However, each profile also proved to be unique–it was the specific combination of MC_N_, verbal, and EF skills, and the correlations among them, that suggest differences in how girls with or without TS approach mathematics during the early school years.

## Supporting information

S1 Table Studies that report mathematics outcomes in girls and adolescents with TS listed in order of publication date. (DOCX)

S1 Text References for S1 Table. (DOCX)
