# Correction: Variation in early number skills and mathematics achievement: Implications from cognitive profiles of children with or without Turner syndrome

**DOI:** 10.1371/journal.pone.0250388

**Published:** 2021-04-13

**Authors:** Sarah L. Lukowski, Emily R. Padrutt, Kyriakie Sarafoglou, Judith L. Ross, Jennifer R. Law, Rachel E. Olson, Michèle M. M. Mazzocco

In the Author Contributions, Sarah L. Lukowski (SLL) should be listed as one of the persons who contributed to Methodology.
