# Supplementary material for: Variation in early number skills and mathematics achievement: Implications from cognitive profiles of children with or without Turner syndrome
Source: PLoS One. 2020 Oct 2;15(10):e0239224. doi: 10.1371/journal.pone.0239224 (PMC7531844; doi:10.1371/journal.pone.0239224)
Supplement: S1 Table — (DOCX) [file pone.0239224.s001.docx]

**S1 Table. Studies that Report Math Outcomes in Girls and Adolescents with TS Listed in Order of Publication Date.**

| **Study** | **Age^a^** | ***N*** |
| --- | --- | --- |
| Pennington et al. [S1] | 7 – 16 | 19 |
| Bender et al. [S2] | 14 – 17 | 9 |
| McCauley et al. [S3] | 9 – 17 | 17 |
| Rovet [S4] | 6 – 16 | 67 |
| Rovet et al. [S5] | 7 – 16 | 45 |
| Mazzocco [S6] | 5 – 16 | 29 |
| Romans et al. [S7] | 13 – 21 | 99 |
| Siegel et al. [S8] | *M* = 10.1 | 22 |
| Temple & Marriott [S9] | 9 – 11 | 11 |
| Mazzocco [S10] | 5 – 6 | 14 |
| Collaer et al. [S11] | 12 + | 21 |
| Temple & Sherwood [S12] | 11 – 12 | 6 |
| Kesler et al. [S13] | 7 – 24 | 15 |
| Mazzocco et al. [S14] | 7 – 11 | 25 |
| Murphy et al. [S15] | 5 – 7 ^b^ | 24 |
| Murphy & Mazzocco [S16] | 10 – 11 | 18 |
| Mazzocco & Hanich [S17] | 5 – 7^b^ | 36 |
| Zougkou & Temple [S18] | 11 – 12 | 2 |
| Attout et al. [S19] | 7 – 33 | 20 |
| Brankaer et al. [S20] | *M* = 9.3 | 24 |
| Baker et al. [S21] | 7 – 15 | 54 |

TS, Turner syndrome.

^a^ Age range in years is reported when available. Mean in years is reported when age range was not reported.

^b^ Participant age at first assessment, a longitudinal sample is reported.
