# Supplementary material for: Variation in early number skills and mathematics achievement: Implications from cognitive profiles of children with or without Turner syndrome
Source: PLoS One. 2020 Oct 2;15(10):e0239224. doi: 10.1371/journal.pone.0239224 (PMC7531844; doi:10.1371/journal.pone.0239224)
Supplement: S1 Text — (DOCX) [file pone.0239224.s002.docx]

**S1 Text. References for S1 Table:**

S1. Pennington BF, Bender B, Puck M, Salbenblatt J, Robinson A. Learning disabilities in children with sex chromosome abnormalities. Child Dev. 1982; doi: 10.2307/1129006

S2. Bender BG, Linden MG, Robinson A. Neuropsychological impairment in 42 adolescents with sex chromosome abnormalities. Am J Med Genet. 1993; doi: 10.1002/ajmg.1320480312

S3. McCauley E, Kay T, Ito J, Treder R. The Turner syndrome: Cognitive deficits, affective discrimination, and behavior problems. Child Dev. 1987; doi: 10.2307/1130523

S4. Rovet JF. The psychoeducational characteristics of children with Turner syndrome. J Learn Disabil. 1993; doi: 10.1177/002221949302600506

S5. Rovet J, Szekely C, Hockenberry M. Specific arithmetic calculation deficits in children with Turner syndrome. J Clin Exp Neuropsychol. 1994; doi: 10.1080/01688639408402696

S6. Mazzocco MMM. A process approach to describing mathematics difficulties in girls with Turner syndrome. Pediatrics. 1998; 102: Supplement 3.

S7. Romans SM, Stefanatos G, Roeltgen DP, Kushner H, Ross JL. Transition to young adulthood in Ullrich-Turner syndrome: Neurodevelopmental changes. Am J Med Genet. 1998; 79(2).

S8. Siegel PT, Clopper R, Stabler B. The psychological consequences of Turner syndrome and review of the National Cooperative Growth Study psychological substudy. Pediatrics. 1998; 102: Supplement 3.

S9. Temple CM, Marriott AJ. Arithmetical ability and disability in Turner’s syndrome: A cognitive neuropsychological analysis. Dev Neuropsychol. 1998; doi: 10.1080/87565649809540700

S10. Mazzocco MMM. Math learning disability and math LD subtypes: Evidence from studies of Turner syndrome, Fragile X syndrome, and Neurofibromatosis Type 1. J Learn Disabil. 2001; doi: 10.1177/002221940103400605

S11. Collaer ML, Geffner ME, Kaufman FR, Buckingham B, Hines M. Cognitive and behavioral characteristics of Turner syndrome: Exploring a role for ovarian hormones in female sexual differentiation. Horm Behav. 2002; doi: 10.1006/hbeh.2001.1751

S12. Temple CM, Sherwood S. Representation and retrieval of arithmetical facts: Developmental difficulties. Q J Exp Psychol A. 2002; doi: 10.1080/02724980143000550

S13. Kesler SR, Menon V, Reiss AL. Neurofunctional differences associated with arithmetic process in Turner syndrome. Cereb Cortex. 2006; doi: [10.1093/cercor/bhj028](https://dx.doi.org/10.1093%2Fcercor%2Fbhj028)

S14. Mazzocco MMM, Singh Bhatia N, Lesniak-Karpiak K. Visuospatial skills and their association with math performance in girls with fragile X or Turner syndrome. Child Neuropsychol. 2006; doi: 10.1080/09297040500266951

S15. Murphy MM, Mazzocco MMM, Gerner G, Henry AE. Mathematics learning disability in girls with Turner syndrome or fragile X syndrome. Brain Cogn. 2006; doi: 10.1016/j.bandc.2005.12.014

S16. Murphy MM, Mazzocco MMM. Mathematics learning diabilities in girls with Fragile X or Turner syndrome during late elementary school. J Learn Disabil. 2008; doi: 10.1177/0022219407311038

S17. Mazzocco MMM, Hanich LB. Math achievement, numerical processing, and executive functions in girls with Turner syndrome: Do girls with Turner syndrome have math learning disability? Learn Individ Differ. 2010; doi: 10.1016/j.lindif.2009.10.011

S18. Zougkou K, Temple CM. The processing of number scales beyond whole numbers in development: Dissociations in arithmetic in Turners syndrome. Cogn Neuropsycol. 2016; doi: 10.1080/02643294.2016.1179178

S19. Attout L, Noel M-P, Nassogne M-C, Rousselle L. The role of short-term memory and visuo-spatial skills in numerical magnitude processing: Evidence from Turner syndrome. PLoS ONE. 2017: doi: 10.1371/journal.pone.0171454

S20. Brankaer C, Ghesquière P, De Wel A, Swillen A, De Smedt B. Numerical magnitude processing impairments in genetic syndromes: a cross-syndrome comparison of Turner and 22q11.2 deletion syndromes. Dev Sci. 2017; doi: 10.1111/desc.12458

S21. Baker JM, Klabunde M, Jo B, Green T, Reiss AL. On the relationship between mathematics and visuospatial processing in Turner syndrome. J Psychiatr Res. 2020; doi: 10.1016/j.jpsychires.2019.11.004
